# Identification of a Serotonin 2A Receptor Subtype of Schizophrenia Spectrum Disorders With Pimavanserin: The Sub-Sero Proof-of-Concept Trial Protocol

**DOI:** 10.3389/fphar.2020.00591

**Published:** 2020-04-30

**Authors:** Olga B. Baltzersen, Herbert Y. Meltzer, Vibe G. Frokjaer, Jayachandra M. Raghava, Lone Baandrup, Birgitte Fagerlund, Henrik B.W. Larsson, H. Christian Fibiger, Birte Y. Glenthøj, Gitte M. Knudsen, Bjørn H. Ebdrup

**Affiliations:** ^1^Centre for Neuropsychiatric Schizophrenia Research (CNSR), Centre for Clinical Intervention & Neuropsychiatric Schizophrenia Research (CINS), Mental Health Centre Glostrup, Glostrup, Denmark; ^2^Departments of Psychiatry and Behavioral Sciences, Pharmacology, and Physiology, School of Medicine, Northwestern University Feinberg School of Medicine, Chicago, IL, United States; ^3^Neurobiology Research Unit and Center for Integrated Molecular Brain Imaging, Copenhagen University Hospital Rigshospitalet, Copenhagen, Denmark; ^4^Mental Health Services Copenhagen, Copenhagen University Hospital Rigshospitalet, Copenhagen, Denmark; ^5^Functional Imaging Unit (FIU), Rigshospitalet Glostrup, Glostrup, Denmark; ^6^Department of Clinical Medicine, Faculty of Health and Medical Sciences, University of Copenhagen, Copenhagen, Denmark; ^7^Department of Psychiatry, University of British Columbia, Vancouver, BC, Canada

**Keywords:** Pimavanserin, antipsychotic-free, first-episode schizophrenia spectrum patients, serotonin 2A receptor positron emission tomography, magnetic resonance imaging, cognition, psychopathology, side-effects

## Abstract

**Background:**

All current approved antipsychotic drugs against schizophrenia spectrum disorders share affinity for the dopamine receptor (D2R). However, up to one-third of these patients respond insufficiently, and in some cases, side-effects outweigh symptom reduction. Previous data have suggested that a subgroup of antipsychotic-naïve patients will respond to serotonin 2A receptor (2AR) blockade.

**Aims:**

This investigator-initiated, translational, proof-of-concept study has overall two aims; 1) To test the clinical effectiveness of monotherapy with the newly approved drug against Parkinson's disease psychosis, pimavanserin, in antipsychotic-free patients with first-episode schizophrenia spectrum disorders; 2) To characterize the neurobiological profile of responders to pimavaserin.

**Materials and Equipment:**

Forty patients will be enrolled in this 6-week open label, one-armed trial with the selective serotonin 2AR antagonist (pimavanserin 34 mg/day). At baseline, patients will undergo: positron emission tomography (PET) imaging of the serotonin 2AR using the radioligand [¹¹C]Cimbi-36; structural magnetic resonance imaging (MRI); MR spectroscopy of cerebral glutamate levels and diffusion tensor imaging; cognitive and psychopathological examinations; electrocardiogram, and blood sampling for genetic- and metabolic analyses.

**Outcome Measures:**

The primary clinical endpoint will be reduction in the Positive and Negative Syndrome Scale (PANSS) positive score. Secondary clinical endpoints comprise multiple clinical ratings (positive and negative symptoms, depressive-, obsessive-compulsive symptoms, quality of life, social functioning, sexual functioning, and side-effects). PET, MRI, and cognitive parameters will be used for in-depth neuropsychiatric characterization of pimavanserin response.

**Anticipated Results:**

Clinically, we expect pimavanserin to reduce psychotic symptoms with similar effect as observed with conventional antipsychotics, for which we have comparable historical data. We expect pimavanserin to induce minimal side-effects. Neurobiologically, we expect psychotic symptom reduction to be most prominent in patients with low frontal serotonin 2AR binding potential at baseline. Potential pro-cognitive and brain structural effects of pimavanserin will be explored.

**Perspectives:**

Sub-Sero will provide unique information about the role serotonin 2AR in antipsychotic-free, first-episode psychosis. If successful, Sub-Sero will aid identification of a “serotonergic subtype” of schizophrenia spectrum patients, thereby promoting development of precision medicine in clinical psychiatry.

**Clinical Trial Registration:**

ClinicalTrials, identifier NCT03994965.

## Introduction

While the pathophysiology of schizophrenia is complex, all currently marketed pharmacological treatment options share dopamine D2 receptor (D2R) affinity. The neurobiological complexity of psychosis is indirectly reflected in the fact that up to one third of the patients with schizophrenia spectrum disorders do not respond sufficiently to dopaminergic drugs. Furthermore, in some patients the side-effects related to D2R antagonism outweigh the symptom reduction.

Clinically, schizophrenia spectrum disorders, such as schizophrenia, schizoaffective disorder, and schizotypy are often associated with life-long symptoms. The clinical features comprise positive psychotic symptoms (e.g. hallucinations and delusions), negative symptoms (e.g. anhedonia and social withdrawal), and marked cognitive deficits. Although current antipsychotics can reduce positive symptoms, the negative symptoms and cognitive deficits are relatively unaffected or may even worsen during treatment (Nørbak-Emig et al., 2016).

D2R antagonism is associated with extrapyramidal side-effects, hyperprolactinemia (Coello et al., 2015), dyslipidemia (Ebdrup et al., 2014), weight gain (Nielsen et al., 2016), as well as subcortical structural brain changes (Ebdrup et al., 2013). Moreover, D2R antagonism may induce tardive dyskinesia and secondary cognitive impairments, and the extrapyramidal side-effects reduce compliance all of which increase the risk of relapse (Meltzer, 2013). Finally, the metabolic side-effect may contribute to the reduced life expectancy of 15–20 years for patients with severe mental disorders (Nordentoft et al., 2013). Considering the severity of these side-effects, ideally, D2R antagonists should only be prescribed to patients, who respond to these drugs. Unfortunately, at present alternative drug treatment strategies are not available. Beyond the shared feature of D2R affinity, most marketed antipsychotics have a more expansive neuro-receptor profile. More specifically, several of the so-called second-generation antipsychotics, e.g. quetiapine, olanzapine, lurasidone, and clozapine are characterized by a relatively low D2R affinity combined with a relatively high serotonin 2AR affinity (Meltzer and Massey, 2011).

In 1957, Carlsson presented evidence that dopamine is a neurotransmitter, and in 1977, he further hypothesized that dopamine hyperfunction may underlie psychotic symptoms in schizophrenia (Carlsson, 1977). The dopamine hypothesis of schizophrenia has been modified over time and the more recent version suggests that schizophrenia patients, who respond to conventional dopamine D2R antagonists, may have an increased dopamine synthesis- and release capacity in the basal ganglia (Howes and Kapur, 2009). Conversely, non-responders to dopamine antagonists may constitute a neurobiologically different subtype.

The involvement of the 5-hydroxytryptamine 2A receptor (serotonin 2AR) system in schizophrenia was proposed in 1954. The hypothesis was based on the observations that lysergic acid diethylamide (LSD) can induce hallucinations and emotional disturbances, which are common clinical features of schizophrenia, and it was discovered that LSD was a potent serotonin 2AR agonist (Woolley and Shaw, 1954).

In 2010, we published the hitherto largest *in vivo* positron emission tomography (PET) study of initially antipsychotic-naïve, first-episode schizophrenia patients, and our findings confirmed the presence of a reduced serotonin 2AR availability in the frontal cortex (Rasmussen et al., 2010). Moreover, before treatment, the cerebral serotonin 2AR availability was inversely associated with the severity of psychotic symptoms (Rasmussen et al., 2010). After treatment with the antipsychotic compound, quetiapine, we found that the reduction in psychotic symptoms was related to the cerebral serotonin 2AR occupancy (Rasmussen et al., 2011). Overall, data from this cohort study supported that a proportion of schizophrenia patients may be responsive to selective serotonin 2AR blockade (Meltzer et al., 2004; Geyer and Vollenweider, 2008). An independent twin study further confirmed that low frontal serotonin 2AR availability may be a state feature of schizophrenia (Rasmussen et al., 2016). Indirect clinical support for a serotonergic target at the earliest disease stage is, that psychosis induced by serotonin 2AR agonism resembles first-episode schizophrenia more than chronic schizophrenia (Geyer and Vollenweider, 2008). Moreover, clozapine, which is the most effective antipsychotic drug, has pronounced antagonism at the serotonin 2AR, and this feature has been suggested to contribute to its superiority (Steward et al., 2004). Finally, genetic studies of serotonin 2A single-nucleotide polymorphism in schizophrenia support the role of the serotonin 2A receptor gene in schizophrenia and the response to antipsychotic drugs (Serretti et al., 2007; Kaur et al., 2017).

In an independent cohort of antipsychotic-naïve patients, we recently provided proof-of-concept to support the notion of subtyping schizophrenia. By means of advanced multimodal and multivariate algorithms on electrophysiological and cognitive data, we identified two distinct subtypes of patients. Before treatment the two subtypes were clinically inseparable, yet their response to selective dopaminergic blockade was predicted with significant accuracy (Bak et al., 2017).

Based on the above, we hypothesize that the clinical effect with monotherapy with a selective 2AR antagonist will be similar to the effect seen with treatment with a D2R antagonist, for which we have comparable, comprehensive historical data. Specifically, we expect psychotic symptom reduction to be most prominent in the patients with the lowest frontal serotonin 2AR binding potential at baseline. With Sub-Sero, we aim to identify a subtype of patient with schizophrenia spectrum disorders, which, if successful, will guide a personalized medicine approach to treating schizophrenia spectrum patients at the earliest stage of the disorder.

## Materials and Equipment

### Study Medication

Pimavansin (Nuplazid™) is a selective serotonin 2AR antagonist with the recent indication of treatment of hallucinations and delusions associated with Parkinson's disease psychosis (FDA approval, April 2016).

Pimavanserin acts as an inverse agonist and antagonist at serotonin 2A receptors with high binding affinity (Ki value 0.087 nM) and at serotonin 2C receptors with lower binding affinity (Ki value 0.44 nM). Pimavanserin has low affinity for sigma 1 receptors (Ki value 120 nM) and has no appreciable affinity (Ki value >300 nM) to dopaminergic (including D2), serotonin 2B, muscarinic, histaminergic, or adrenergic receptors, or to calcium channels. The mean plasma half-lives for pimavanserin and the active metabolite (N-desmethylated metabolite) are approximately 57 and 200 h, respectively. This results in stable plasma concentrations with only one tablet a day. The median time to maximum concentration (T_max_) of pimavanserin is 6 h and independent of dose. Absorption is independent of meal ingestion. In human plasma, the distribution pimavanserin is predominantly protein bound (~95%) (Vanover et al., 2007).

### Side-Effects

Side-effects in placebo-controlled studies of 6-week treatment (N=202) comprise nausea 7%, constipation 4%, peripheral oedema 7%, gait disturbance 2%, hallucination 5%, and confusion 6% (Vanover et al., 2007). After approval of pimavanserin, somnolence, rash, urticaria, and reactions consistent with angioedema (e.g., tongue swelling, circumoral oedema, throat tightness, and dyspnoea) have only been sporadically reported (Fda, 2017).

Pimavanserin prolongs the QT interval, and although no other ECG abnormalities have been linked to pimavanserin, treatment should be avoided in patients with known QT prolongation or in combination with other drugs known to prolong QT interval (Ballard et al., 2018).

### Clinical Data on Pimavanserin

We conducted a literature search on [pimavanserin AND schizophrenia] in the databases PubMed and ClinicalTrials for the results of studies on pimavanserin and the effect on schizophrenia spectrum patients (search date: July 16^th^, 2019). The PubMed search resulted in 26 hits: 6 reviews on possible use in schizophrenia; 8 hits related to Parkinson's Disease; one commentary article, and the 2 original papers, which are summarized below.

The ClinicalTrials search resulted in 14 hits. Besides the current study protocol and the study by Meltzer et al. mentioned below, three studies are currently investigating the effect of pimavanserin as add on therapy for schizophrenia patients (NCT03121586; NCT02970292; NCT02970305). All are initiated by the manufacturer ACADIA Pharmaceuticals. The ten remaining studies were in patients with Parkinson's and Alzheimer's diseases. The current Sub-Sero trial (NCT03994965) will be the first study to use pimavanserin as monotherapy in schizophrenia spectrum patients.

In 2012, Meltzer et al. published a randomized double blinded trial that examined the potential efficacy of pimavanserin as an add-on treatment to risperidone and haloperidol, respectively, compared with risperidone as monotherapy (Meltzer et al., 2012). The study was conducted on 423 patients with chronic schizophrenia and a recent exacerbation of psychotic symptoms. Patients were randomized in four groups and treated for 6 weeks of risperidone 2 mg + placebo, risperidone 2 mg + pimavanserin 20 mg, risperidone 6 mg + placebo, haloperidol 2 mg + placebo, or haloperidol 2 mg + pimavanserin 20 mg. The reduction in Positive and Negative Syndrome Scale (PANSS) total score with risperidone 2 mg + pimavanserin by end of study was significantly greater than risperidone 2 mg + placebo: −23.0 vs. −16.3 (p=0.007), and there was no significant difference from the risperidone 6 mg + placebo group. Weight gain and hyperprolactinemia were greater in the risperidone 6 mg + placebo group than the risperidone 2 mg + pimavanserin group, but there was no difference in extrapyramidal side-effects. Overall, the study supported preclinical data (Gardell et al., 2007) indicating that serotonin 2A antagonism has potential as add-on treatment, which may enable dose reduction of conventional antipsychotics.

In 2019, Nasrallah et al. published an open label case series of 10 patients with refractory psychosis successfully treated with add-on pimavanserin to their usual treatment (Nasrallah et al., 2019). Six of the patients had previously tried, but not responded to clozapine. All patients showed marked response to augmentation with pimavanserin 34 mg/day within 4–8 weeks. Improvements of negative symptoms and social functioning were also reported. None of the patients discontinued pimavanserin due to side-effects. Despite the open label, non-controlled design, these cases provide encouraging perspectives for usage of pimavanserin in schizophrenia patients and importantly pimavanserin appeared well-tolerated with a favorable side-effect profile.

## Methods

### Study Design

One-armed, open label, investigator-initiated, proof-of-concept trial.

### Study Population

#### Patients

We will include antipsychotic-free [no prior use of antipsychotics longer than two weeks in the previous year or 6 weeks lifetime, or antipsychotic treatment within 30 days prior to inclusion, same criteria as the OPTiMiSE cohort (Kahn et al., 2018)], first-episode schizophrenia spectrum patients. ICD-10 diagnoses are shown in **Table 1**. Patients will be recruited from psychiatric hospitals and outpatient psychiatric centers in the capital region (Copenhagen area). Before inclusion, diagnoses will be verified by schedules for clinical assessment in neuropsychiatry (SCAN) (Wing et al., 1990). In- and exclusion criteria are shown in **Table 1**.

**Table 1:** In- and Exclusion Criteria in Sub-Sero.

Inclusion criteria
Antipsychotic-free^1^
Fulfilling diagnostic criteria of schizophrenia, persistent delusional disorder, acute and transient psychotic disorders, schizoaffective disorder, other non-organic psychotic disorders and unspecified non-organic disorders (ICD-10: F20.x; F22.x; F23.x; F24.x; F25.x; F28; F29)
Age: 18-45 years
Legally competent

**Exclusion criteria**
Current substance dependence ICD-10 (F1x.2) or substance abuse in any period up to 3 months prior to referral (exception: tobacco/nicotine, F17.2)
Head injury with more than 5 minutes of unconsciousness
Any coercive measure
Metal implanted by operation
Pacemaker
Pregnancy (assessed by urine HCG)
Female patients: Unwillingness to use safe contraception (Intra Uterine Device/System or hormonal contraceptives) during the study period including the wash out period.
Severe physical illness
Known QT prolongation or congenital prolongation of the QT interval
Medical history of cardiac arrhythmias, as well as other circumstances that may increase the risk of torsade de pointes and/or sudden death, including symptomatic bradycardia, hypopotassemia or hypomagnesemia
Current treatment with drugs known to prolong QT interval^2^
Allergies to any of the inactive ingredients and film coat components^3^

^1^No prior use of antipsychotic medication longer than an episode of two weeks in the previous year and/or 6 weeks lifetime, and/or antipsychotic treatment within 30 days prior to inclusion (Kahn et al., 2018).

^2^Class 1A antiarrhythmics (e.g., quinidine, procainamide); Class 3 antiarrhythmics (e.g., amiodarone, sotalol); certain antibiotics (e.g., gatifloxacin, moxifloxacin).

^3^Pregelatinized starch, magnesium stearate, microcrystalline cellulose, hypromellose, talc, titanium dioxide, polyethylene glycol, and saccharin sodium.

#### Trial Visits and Examinations

The patients will undergo thorough examinations on three overall domains: 1) clinical ratings and assessments; 2) neurocognitive testing; 3) neuroimaging. The examination program is shown in **Table 2**.

**Table 2:** Examination Program of the Sub-Sero Study.

		PATIENTS (N=40)	
		Clinical	Imaging
**Baseline**	**Week 0**	SCAN interview Cognition Blood, ECG, BMI PANSS, BNSS, CDSSSWN, UKU, BOCS, EHI, CGIPSP, GAF, QLS, CSFQ	PET, [¹¹C]Cimbi-36 MRS sMRI/DTI
**Treatment**			
	**Week 1**	**Pimavanserin** (34 mg/day)PANSS, ECG Blood, weight BNSS, CDSS SWN, UKU, BOCS, PSP	
	**Week 2**	PANSS, ECG Blood, weight BNSS, CDSS SWN, UKU, BOCS, PSP	–
	**Week 4**	PANSS, ECG Blood, weight BNSS, CDSS SWN, UKU, BOCS, PSP	–
	**Week 6**	Cognition Blood, ECG, BMI PANSS, BNSS, CDSSSWN, UKU, BOCS, PSP, GAF, QLS, CGI, CSFQ	MRS sMRI/DTI
**Wash out**	**Week 8**	Blood, ECG PANSS	–

SCAN, Schedules for clinical assessment in neuropsychiatry; PANSS, Positive and negative syndrome scale; BNSS, Brief Negative Symptom Scale; BNSS, Calgary depression scale for schizophrenia; BOCS, The Brief Obsessive Compulsive Scale; SWN, Subjective well-being under neuroleptic treatment scale; UKU (measurement of side-effects), Udvalg for Kliniske Undersøgelser; PSP, Personal and Social Performance Scale; EHI, Edinburgh Handedness Inventory; BMI, Body Mass Index; QLS, Quality of Life Scale; CSFQ, Changes in Sexual Functioning Questionnaire; MRS, Magnetic resonance spectroscopy; sMRI, structural Magnetic Resonance Imaging; DTI, Diffusion Tensor Imaging.

#### Clinical Ratings

Symptom severity will be assessed by the PANSS (Kay et al., 1989). Moreover, negative symptoms will be assessed using the Brief Negative Symptom Scale (BNSS) (Kirkpatrick et al., 2011). Depressive symptoms of will be assessed using the Calgary Depression Scale for Schizophrenia (CDSS) (Addington et al., 1993). Symptoms of obsessive compulsive character will be assessed with The Brief Obsessive Compulsive Scale (BOCS) (Bejerot et al., 2014). The Subjective well-being under neuroleptic treatment scale (SWN) (Naber et al., 2001) will used as measure for self-rated wellbeing during treatment and life quality will be assessed with the Quality of Life Scale (QLS) (Heinrichs et al., 1984). Level of functioning will be assessed by the Personal and Social Performance Scale (PSP) (Morosini et al., 2000). We will use the Changes in Sexual Functioning Questionnaire (CSFQ) (Clayton et al., 1997) to asses sexual functioning and side-effects. Handedness will be assessed with Edinburgh Handedness Inventory (EHI) (Oldfield, 1971).

#### Neuropsychiatric Testing

The Cambridge Neuropsychological Test Automated Battery (CANTAB) (Robbins et al., 1994) will be used to assess multiple cognitive domains: working memory, learning and executive functions; visual, verbal, and episodic memory; attention, information processing, and reaction time; social and emotion recognition, decision making and response control. Premorbid IQ will be tested using the Danish version of the National Adult Reading Test (DART) (Hjorthoj et al., 2013) (adapted from the National Adult Reading Test, NART) (Nelson, 1982), and the current intelligence will be assessed with the WAIS-IV battery (Kreutzer et al., 2011).

### Neuroimaging

#### Positron Emission Tomography (PET)

PET-scans using the radioligand tracer [¹¹C]Cimbi-36 will be conducted at Neurobiology Research Unit at Rigshospitalet in order to estimate the cerebral serotonin 2A receptor binding potential. The serotonin 2AR agonist PET radioligand [¹¹C]Cimbi-36 will be produced for human administration, as previously described (Ettrup et al., 2014). All participants will be scanned at baseline in a high-resolution research tomography (HRRT) PET scanner (CTI/Siemens, Knoxville, TN).

PET will not be repeated after 6 weeks. Instead, the relation between plasma pimavanserin and cerebral serotonin 2AR occupancy will be based on data obtained in 5 healthy volunteers: [¹¹C]Cimbi-36 PET will be performed before intake of a single dose of pimavanserin 34 mg. PET will be repeated twice within the next two days to determine the relation between cerebral serotonin 2AR occupancy and plasma-pimavanserin. By measuring patients' plasma- pimavanserin levels after 6 weeks, when in steady-state, we will use this plasma-pimavanserin/2AR occupancy ratio derived from the healthy controls to index patients' serotonin 2AR occupancy.

In order to compare baseline serotonin 2AR binding potential, patients will be compared with a group of comparable healthy historical controls balanced on age and gender from the CIMBI data-base (Knudsen et al., 2016).

#### Magnetic Resonance Imaging (MRI)

We will use a Philips Achieva 3.0 T whole-body MRI scanner (Philips Healthcare, Best, The Netherlands) with a 32 channel SENSE Head Coil (Invivo, Orlando, Florida, USA) at the Functional Imaging Unit, Rigshospitalet-Glostrup. The MRI scans will be used for co-registration of PET imaging. In addition, T1- and T2 weighted structural images, diffusion tensor imaging (DTI), and spectroscopy (MRS) sequences will be performed, in order to explore cortical and subcortical brain structures, the integrity of white matter tracts and thalamic glutamate levels before and after treatment.

### Intervention

#### Treatment Regimen

Patients will be treated in monotherapy with tablet pimavanserin (Nuplazid^®^) 34 mg/day, oral administration for 6 weeks. At the end of study there will be a washout period of two weeks. The washout procedure after 6 weeks will be similar to the procedure for switching to rescue medication (described below). At week 8, the patients will be seen for their last follow-up visit. The pimavanserin tablets will be provided by ACADIA Pharmaceuticals and re-packed and re-labelled by “Region Hovedstadens Apotek” according to Danish legislation and good clinical practice (GCP) regulations. No placebo medication will be administered in this study.

Tolerability to pimavanserin and clinical effect will be monitored closely. Intolerable side-effects or clinical worsening (defined as a 20% increase in PANSS positive subscale score compared to baseline PANSS positive score) will result in switching to rescue medication.

#### Rescue Medication

-As primary rescue medication, we will use a benzodiazepine rather than a conventional antipsychotic compound, due to the risk of QTc prolongation after pimavanserin exposure (pimavanserin T½= 57 h). We will use oxazepam at per needed basis: 15–30 mg up to 3 times daily.

-As secondary rescue medication, we will commence conventional antipsychotic treatment with amisulpride (Solian™). Amisulpride is a selective dopamine D2/3 receptor antagonist (50–800 mg/day) and is a first-line compound to treat schizophrenia spectrum disorders in Denmark. Amisulpride will be initiated 7 to 14 days after the switch to oxazepam. The washout period of pimavanserin is estimated based on the assumption that T½= 57 h, and the recommended washout period for a complete washout is 5–10 × T½ (Nelson, 1982) [T½ pimavanserin = 57 h: (5x57h)/24 = 11.9 days]. For the current study, a complete washout is not essential as long as ECG, i.e. the QTc interval, is normal (< 500 msec). Since amisulpride will only be initiated if ECG is normal, we judge a washout period of 7–14 days enough to balance the patient's need of treatment with respect to cardiac safety on the one hand and minimizing side-effects of prolonged benzodiazepine exposure on the other hand.

### Risks and Potential Benefits

Currently, pimavanserin only has the indication of hallucinations and delusions associated with Parkinson's disease psychosis. Hence, in Sub-Sero, we will use pimavanserin *off-label*. We believe that pimavanserin in monotherapy holds promise as an alternative new treatment option to conventional antipsychotic medication in patients with schizophrenia spectrum disorders. Thus, in despite of the fact that the drug will be used off-label, we believe that the known side-effects of pimavanserin will be markedly milder than those of conventional antipsychotic medication. In particular, the risks of extrapyramidal symptoms (EPS) and hyperprolactinemia are limited with pimavanserin (Robinson et al., 2005).

No interim analyses will be performed. Instead the open label design permits a continuous evaluation of potential side-events and the overall safety of the study. Patients will be monitored closely by the study physicians and nurses, and side-events will be reported to the Danish Medical Authorities through an electronic report system. The study will be monitored closely by GCP throughout the study period.

By participating in Sub-Sero, patients will undergo more comprehensive testing, scans, and examinations than if undergoing treatment in a conventional regional mental health setting. Although this examination program may be burdensome for some patients, the thorough examinations may also be viewed as a potential benefit.

### Outcome Measures and Analysis Methods

#### Primary Endpoint

The primary clinical endpoint will be reduction in the PANSS positive subscale score [PANSS positive baseline - PANSS positive at week 6].

#### Secondary Endpoints

Secondary clinical endpoints include reduction in PANSS positive subscale score compared to baseline by week 2 and 4, respectively. Also, we will explore effects on negative, obsessive symptoms, sexual functioning, as well as the proportion of patients achieving symptomatic remission (Andreasen criteria) at week 6 (Robbins et al., 1994).

Secondary neuropsychiatric endpoints comprise in-depth characterization pre-treatment predictors of symptom reduction. Measurements will comprise cerebral serotonin 2AR binding potential, cognition, thalamic glutamate levels, blood test, and genetic analyses. After 6 weeks, also the degree of 2AR occupancy in patients, as indexed by plasma-pimavanserin, will be determined.

## Anticipated Results

### Sample Size Calculation

In Sub-Sero, we hypothesize that treatment with pimavanserin will have a similar clinical effect as was observed in a previous, comparable cohort in which patients underwent 6 weeks of treatment with amisulpride. Neurobiologically, we expect psychotic symptom reduction after pimavanserin treatment to be most prominent in patients with low frontal serotonin 2AR binding potential at baseline.

**Figure 1:**
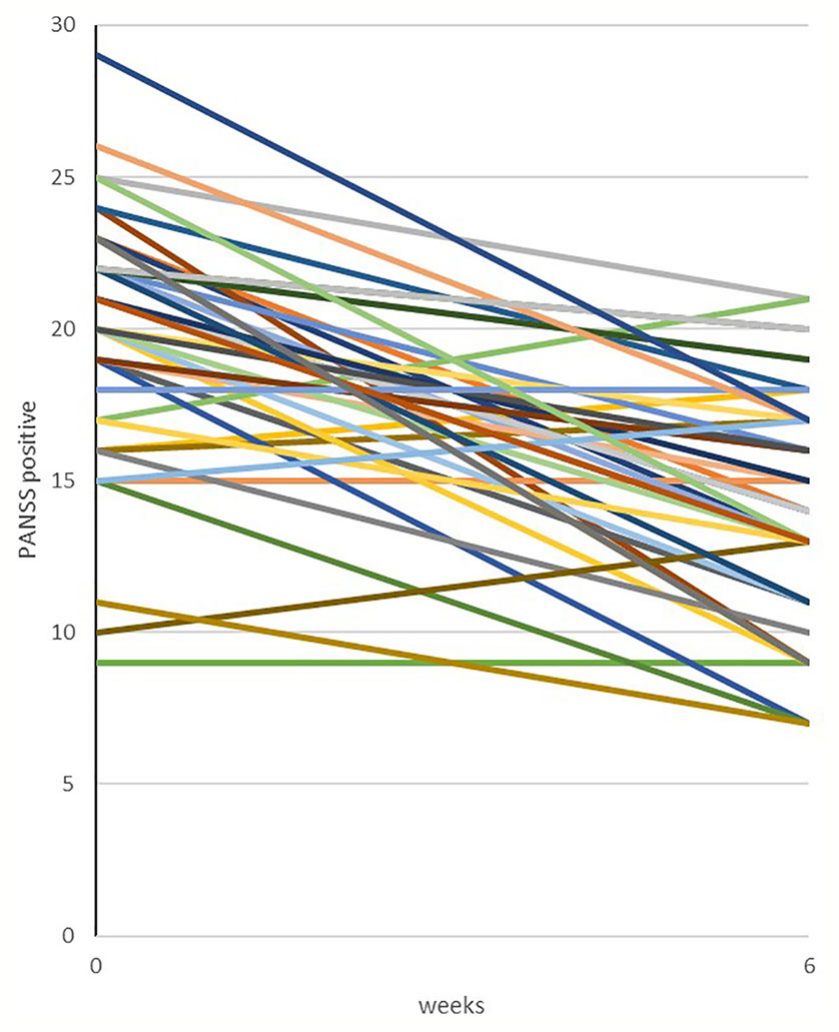
Treatment response, amisulpride. Historical data from our group showing changes in PANSS positive symptoms in an independent cohort of 46 antipsychotic-naïve, first-episode schizophrenia patients treated with amisulpride (mean dose of 279.4 mg/day) monotherapy for six weeks. Details on the cohort have previously been published, e.g.(Nielsen et al., 2012a; Nielsen et al., 2012b; Nielsen et al., 2016; Wulff et al., 2019).

Our previous amisulpride cohort comprised 46 initially antipsychotic-naïve, first-episode schizophrenia patients. Details on the cohort are provided in, e.g. (Nielsen et al., 2012a; Nielsen et al., 2012b; Nielsen et al., 2016; Wulff et al., 2019). The 6 weeks of treatment with amisulpride, a dopamine D2R/D3R antagonist [with serotonin 7, but not serotonin 2A affinity (Abbas et al., 2009)], produced a mean reduction in PANSS positive score of 5.98 with a standard deviation of 4.68 (**Figure 1**). We have estimated a “trial effect” (or natural variation) to a reduction in PANSS positive to 2.5. With 46 patients treated with amisulpride for 6 weeks, the observed power was 95%.

We have used these effect estimates in our sample size calculation for the Sub-Sero. Thus, when setting the desired power to 80% and the significance level of a=0.05, the needed sample size for Sub-Sero is 29 patients completing 6 weeks of pimavanserin treatment.

In our previous cohort, the attrition rate was 25%. Assuming a similar attrition rate of 25% in Sub-Sero, we aim to include 40 patients at baseline. In order to secure power in Sub-Sero, we will continue inclusion of patients until 30 patients have completed 6 weeks follow-up examinations.

### Hypotheses

- Patients treated with pimavanserin in monotherapy will display a comparable response as patients treated with a conventional dopamine D2 antagonist (amisulpride). Observed response to 6 weeks of amisulpride treatment is shown in **Figure 1** and is based on comprehensive historical data from our group (**Figure 1**).
- Pimavanserin will be well-tolerated and induce fewer side-effects (e.g. fewer EPS, minimal weight gain and minimal hyperprolactinemia) than amisulpride (compared with historical data).
- Neurobiologically, we expect psychotic symptom reduction to be most prominent in the patients with the lowest frontal serotonin 2AR binding potential at baseline.
- Pimavanserin will produce improvements in measures of attention, verbal fluency, executive function, and working memory.
- Pimavanserin will exert non-detrimental effects on brain structure.

## Discussion

We anticipate that the Sub-Sero trial will contribute novel clinical and neuropsychiatric data from a cohort of antipsychotic-free, first-episode schizophrenia spectrum patients exposed to monotherapy with a selective 2AR antagonist.

The open-label study design of Sub-Sero is motivated by the fact that pimavanserin will be used off-label for a vulnerable group of antipsychotic-free, first-episode patients with schizophrenia spectrum disorder, who are challenging to recruit. Moreover, the current sparse level of evidence for efficacy of pimavanserin in this patient group remains to be demonstrated before embarking on a randomized controlled trial (RCT) design. Since clinical relevance is the highest priority in Sub-Sero, we chose psychotic symptom reduction as our primary outcome. We have no active control group, but the open label design permits us to compare treatment response with our previous cohorts, on which we have comparable and comprehensive data, e.g. see **Figure 1**.

Inherently, Sub-Sero should not be regarded a pivotal clinical trial. To truly estimate treatment effect of pimavanserin a double-blinded RCT is necessary. We argue that the burden of the disease in these vulnerable patients, along with the current insufficient treatment options justify this proof-of-concept trial with pimavanserin monotherapy.

We expect that Sub-Sero will provide unique information of the role serotonin 2AR in antipsychotic-free, first-episode psychosis, which may inform the design of future randomized clinical trials testing effectiveness of pimavanserin as monotherapy. If successful, Sub-Sero will aid identification of a “serotonergic subtype” of schizophrenia spectrum patients, thereby promoting the development of precision medicine for use in clinical psychiatric practice.

## Publication and Media

Positive and negative results will be published in international, scientific peer-reviewed journals, and data will be presented at national and international conferences.

## Author Contributions

BE conceived the concept of the study, and the study design was adjusted in discussions with HM, CF, BG, and GK. OB, VF, JR, BF, LB, and HL refined the design with respect to their respective fields of expertise. OB and BE drafted the manuscript. All authors approved the submitted version of the manuscript.

## Funding

Economic support for the Sub-Sero trial has been applied for at several non-commercial foundations. ACADIA Pharmaceuticals, which is the sole manufacturer of pimavanserin, has agreed to provide pimavanserin tablets for the Sub-Sero trial. ACADIA Pharmaceuticals had no influence on study design and will not be involved in data processing or in publishing the results of the trial. Centre for Clinical Intervention & Neuropsychiatric Schizophrenia Research (CINS) is funded by an independent grant from the Lundbeck Foundation (R155-2013-16337).

## Conflict of Interest

HM is a consultant to and grantee of ACADIA; he has received support from Eli Lilly, Allergan, Takeda, Sunovion, Dainippon Sumitomo. VF has received lecture fees and honorarium as consultant for Lundbeck Pharma A/S and Sage Therapeutics. BE has received lecture fees and/or is part of Advisory Boards of Bristol-Myers Squibb, Eli Lilly and Company, Janssen-Cilag, Otsuka Pharma Scandinavia AB, Takeda Pharmaceutical Company and Lundbeck Pharma A/S.

The remaining authors declare that the research was conducted in the absence of any commercial or financial relationships that could be construed as a potential conflict of interest.
